# Dopaminergic- and Serotonergic-Dependent Behaviors Are Altered by Lanthanide Series Metals in *Caenorhabditis elegans*

**DOI:** 10.3390/toxics12100754

**Published:** 2024-10-17

**Authors:** Anthony Radzimirski, Michael Croft, Nicholas Ireland, Lydia Miller, Jennifer Newell-Caito, Samuel Caito

**Affiliations:** 1 Department of Pharmaceutical Sciences, School of Pharmacy, Husson University, Bangor, ME 04401, USA; 2 Department of Molecular & Biomedical Sciences, University of Maine, Orono, ME 04469, USAjennifer.newellcaito@maine.edu (J.N.-C.)

**Keywords:** dopamine, serotonin, lanthanum, cerium, erbium, ytterbium

## Abstract

The lanthanide series elements are transition metals used as critical components of electronics, as well as rechargeable batteries, fertilizers, antimicrobials, contrast agents for medical imaging, and diesel fuel additives. With the surge in their utilization, lanthanide metals are being found more in our environment. However, little is known about the health effects associated with lanthanide exposure. Epidemiological studies as well as studies performed in rodents exposed to lanthanum (La) suggest neurological damage, learning and memory impairment, and disruption of neurotransmitter signaling, particularly in serotonin and dopamine pathways. Unfortunately, little is known about the neurological effects of heavier lanthanides. As dysfunctions of serotonergic and dopaminergic signaling are implicated in multiple neurological conditions, including Parkinson’s disease, depression, generalized anxiety disorder, and post-traumatic stress disorder, it is of utmost importance to determine the effects of La and other lanthanides on these neurotransmitter systems. We therefore hypothesized that early-life exposure of light [La (III) or cerium (Ce (III))] or heavy [erbium (Er (III)) or ytterbium (Yb (III))] lanthanides in *Caenorhabditis elegans* could cause dysregulation of serotonergic and dopaminergic signaling upon adulthood. Serotonergic signaling was assessed by measuring pharyngeal pump rate, crawl-to-swim transition, as well as egg-laying behaviors. Dopaminergic signaling was assessed by measuring locomotor rate and egg-laying and swim-to-crawl transition behaviors. Treatment with La (III), Ce (III), Er (III), or Yb (III) caused deficits in serotonergic or dopaminergic signaling in all assays, suggesting both the heavy and light lanthanides disrupt these neurotransmitter systems. Concomitant with dysregulation of neurotransmission, all four lanthanides increased reactive oxygen species (ROS) generation and decreased glutathione and ATP levels. This suggests increased oxidative stress, which is a known modifier of neurotransmission. Altogether, our data suggest that both heavy and light lanthanide series elements disrupt serotonergic and dopaminergic signaling and may affect the development or pharmacological management of related neurological conditions.

## 1. Introduction

Rare earth elements (REEs) are comprised of 17 f-block inner transition elements of the periodic table, which include the lanthanide series (such as lanthanum (La), cerium (Ce), erbium (Er), gadolinium (Gd), and ytterbium (Yb)), along with scandium (Sc) and yttrium (Y)]. REEs and REE-containing alloys are used in many technological devices, such as cell phones, computers, and tablets; rechargeable batteries; fertilizers; antimicrobials; contrast agents for medical imaging; and fuel additives [1,2,3,4]. Lanthanides can reach aquatic ecosystems by exhaust emissions, agricultural leaching, and via effluents from waste water treatment plants from industry [5]. Levels of REEs have risen significantly in both industrial areas and in areas that do not contain lanthanide-utilizing sites [6,7,8,9]. Since all electronic devices have a finite lifespan, devices containing REEs are eventually disposed as electronic waste (e-waste). Studies have shown that the closer in proximity a person is to areas of REE mining, e-waste burning, and e-waste dismantling or processing plants, the higher the level of lanthanides (or other REEs) present in their body, as measured by scalp hair metal content [10,11,12]. Lanthanides have been found to be present in electronic cigarettes (e-cigarettes) at levels significantly higher than traditional cigarettes [13]. Studies have shown that the longer one uses e-cigarettes, the higher the lanthanides accumulate in the body [13]. Additionally, over the past three decades, Ce- and La-based catalytic additives have been used for diesel fuel stabilization. Individuals who repair and maintain diesel engines or are around diesel exhaust are exposed to lanthanides on a regular basis [4,14].

While the use of lanthanide elements is on the rise, their effects on human health are not fully characterized. The use of lanthanide-containing medicines and contrast agents has revealed that lanthanides can be neurotoxic. Lanthanum carbonate is used as a phosphate binder to treat hyperphosphatemia in end-stage kidney disease. Hallmark signs of lanthanum carbonate overdose are both gastrointestinal and neurological symptoms. The neurological symptoms range from headaches to more serious symptoms such as seizures and encephalopathy [15,16,17,18]. There is a growing body of evidence examining the neurotoxicity of Gd-based contrast agents (GBCAs). GBCAs are intravenous drugs used in imaging procedures to enhance the quality of magnetic resonance imaging (MRI) and magnetic resonance angiography (MRA). GBCAs enter the brain via the cerebrospinal fluid (CSF) [19]. Case reports of patients that accidentally receive too high a dose of GBCAs demonstrate that these agents can cause severe headaches, confusion, aphasia, rigidity, visual disturbances, and fatal encephalopathy [20,21,22]. Mechanisms that underlie these symptoms remain to be determined.

Mechanistic studies performed in rodents or in vitro experiments in cell lines have revealed that lanthanides can disrupt multiple neurotransmitter systems. In rodents, La has been shown to impair learning and memory, damage the hippocampus, activate microglia, and disrupt the blood–brain barrier (BBB) [23,24,25,26,27]. BBB disruption has been shown in endothelial bEnd.3 cells by a calcium-dependent rearrangement of actin cytoskeleton and loss of cadherins [28]. Early-life exposure to La impairs behavior and cognition in adult rats [24,25]. Similarly to La, Ce has been shown in rodents to damage the hippocampus, inhibit neural stem cells, disrupt neurotransmitter signaling, and decrease thyroid-stimulating hormone levels [29,30,31]. Adult mice exposed to CeCl_3_ for 60 days had dose-dependent impairments of behaviors including spatial recognition memory as well as decreased neurotransmitters, including dopamine (DA) [32]. DA and noradrenaline release were shown to be significantly decreased in rat brain synaptosomes exposed to lanthanum [33]. Rats exposed in utero to La had significantly decreased levels of serotonin (5-HT) and acetylcholine, but increased levels of glutamate in the brain [25]. In addition to the levels of neurotransmitters being decreased, 5-HT-induced currents through 5-HT receptors were found to be suppressed by La exposure in mouse neuroblastoma N1E-115 cells [34]. La and europium (Eu) have also been shown to inhibit 5-HT transporters in JAR human placental choriocarcinoma cells [35]. However, as these are placental cancer cells, it is unknown if there are similar effects on 5-HT transporters in neurons. La(NO_3_)_3_ was shown to alter DA, glutamate, GABA, and serotonin neurotransmitters in *C. elegans* [36]. La(NO_3_)_3_ is a compound used in the extraction and purification of lanthanum from its ores. It is unknown how other lanthanide elements may affect DA or 5-HT neurosignaling in *C. elegans*. We hypothesized that the dopaminergic (DAergic) and serotonergic neuronal function would be diminished by La and Ce in *C. elegans* and that lanthanides of higher atomic mass, such as erbium (Er) and ytterbium (Yb), would also disrupt these neurotransmitter systems. As previously discussed, La and Ce have been extensively studied due to their use in industry. Er and Yb were selected as heavier lanthanides for the study due to their increasing use in industry and medical and dental applications [37,38,39,40,41,42]. To test this hypothesis, we exposed worms to lanthanum (III) chloride (LaCl_3_), cerium (III) chloride (CeCl_3_), erbium (III) chloride (ErCl_3_), or ytterbium (III) chloride (YbCl_3_) and tested for lethality, DA and 5-HT contents, and DA- and 5-HT-dependent behaviors. Furthermore, we investigated the ability of the four lanthanide elements to induce oxidative stress in the worms, a major determinant in neurotoxicity. This was performed by measuring reactive oxygen species (ROS), advanced glycation end product (AGE) adduct formation on proteins, glutathione content, antioxidant response element activation, and ATP production.

## 2. Materials and Methods

### 2.1. Reagents

Unless otherwise stated, all reagents were obtained from Sigma-Aldrich (St. Louis, MO, USA). LaCl_3_ > 99.5% purity (Molecular weight: 371.37, CAS Number: 10025-84-0, Thermo Scientific Chemicals, Ward Hill, MA, USA), CeCl_3_ > 99% purity (Molecular Weight: 372.58, CAS Number: 18618-55-8, BeanTown Chemical, Hudson, NH, USA), ErCl_3_ > 99.9% purity (Molecular weight: 273.61, CAS Number: 19423-85-9, Thermo Scientific Chemicals), and YbCl_3_ > 99.9% purity (Molecular weight: 279.40, CAS Number: 19423-87-1, Thermo Scientific Chemicals).

### 2.2. C. elegans Strains and Handling of the Worms

*C. elegans* strains were handled and maintained at 25 °C on Nematode Growth Medium (NGM) plates seeded with OP50 strain of *Escherichia coli*, as previously described [43]. The following strains were used in this study: wild-type N2, VP596 (dvls19[pAF15(gst-4::GFP [green fluorescent protein]::NLS)]; vsls33[dop-3::RFP (red fluorescent protein)], and PE254 (fels4[Psur-5::luc+::gfp; rol-6 (su1006)]V). All strains were provided by the *Caenorhabditis* Genetic Center (CGC; University of Minnesota). Eggs were harvested from gravid populations of worms to synchronize strains to the same developmental stage (L1). Synchronization was performed by using a bleaching solution (1% NaOCl and 0.25 M NaOH) followed by a sucrose gradient to segregate eggs from worm and bacterial debris [44]. L1 worms were then treated with various lanthanide concentrations for 30 min in NaCl (85 mM) at 25 °C on an Eppendorf tube rotator. L1s were treated with the lanthanides as L1 stage is the most sensitive to metals and other chemical compounds [45,46,47]. Treated worms were aged to L4 or older for experiments that required older life stages.

### 2.3. Dose–Response Curves

The lethal dose (LD_50_) of La (III), Ce (III), Er (III), and Yb (III) was determined by treating 5000 synchronized L1 worms with doses ranging from 0 to 50 mM for 30 min in 85 mM NaCl at 25 °C on a microcentrifuge tube rotator. All exposures were carried out in triplicate and repeated 5 times. After treatment, worms were washed 3 times with 85 mM NaCl, transferred to OP50-seeded NGM plates, and manually counted for lethality 24 h post-treatment. Calculation of the LD_50_ was performed using Prism 8.4.3 software.

### 2.4. Dopamine-Dependent Behaviors

For each DA-dependent behavior tested, 20 worms per condition were evaluated per experiment, with each experiment being repeated five times. *Basal Slowing Response (BSR)*. The BSR assay was used to measure DA-dependent behavior that mediates the worm’s slowing movement to consume food. The basal slowing response assay was performed as previously described [48]. The number of forward-directed body bends was scored for worms 72 h post-lanthanide treatment placed either on NGM-seeded or -unseeded plates with OP50 *E. coli*. Data are presented as the change in body bends, which are calculated by subtracting the number of body bends of worms plated on *E. coli*-seeded plates from the number of body bends of worms plated on unseeded plates. *Swim-to-crawl Transition*. The transitioning between swimming and crawling in *C. elegans* is controlled by the DAergic nervous system. Swim-to-crawl onset assay was performed as previously described [49]. Seventy-two hours post-lanthanide treatment, worms were placed in a 5 μL M9 droplet on an unseeded NGM plate. Individual worms were manually watched. Measurement of the transition time was started when the worm had reached the M9 droplet edge. The timer was stopped once the worm exited the M9 droplet and was sinusoidal crawling on the agar. *Egg laying*. The egg-laying behavior is representative of both DA and serotonin (5-HT) neuronal function [50]. Eggs were quantified from both inside the treated worms and eggs that were laid on the plate, and the percentage of the total eggs that were laid was calculated. Thirty hours after treatment, upon reaching L4 stage, individual worms were placed on an NGM plate seeded with OP50 *E. coli*. After egg laying occurred, the number of eggs on the agar were counted as were the eggs remaining in the worm. Data are expressed as eggs laid as a percent of the total number of eggs per worm.

### 2.5. Serotonin-Dependent Behaviors

For each 5-HT-dependent behavior tested, 20 worms per condition were evaluated per experiment, with each experiment being repeated five times. *Luminescence Pharynx Pump Rate.* 5-HT regulates the nematode pharynx muscles, by modulating cholinergic and glutamatergic motor neurons on pharyngeal muscle cells [51]. The pharynx pump rate was assessed using PE254 worm strain (fels4[Psur-5::luc+::gfp; rol-6 (su1006)]V), which expresses luciferase in the pharyngeal muscles, as previously described [52]. L1 PE254 worms were treated with lanthanides plated on NGM plates, and pharyngeal pump rate was assessed 72 h later. Worms were loaded into wells of a white 96-well microtiter plate containing 20 g/L OP50 *E. coli* in S Basal medium and 200 μM D-luceiferin. Luminescence was read every 5 min for 1 h. Data are reported as rate of luminescence per hour. *Crawl-to-swim Transition*. The transitioning from crawling to swimming in *C. elegans* is controlled by the serotonergic nervous system. Crawl-to-swim onset assay was performed as previously described [49]. Seventy-two hours post-lanthanide treatment, worms were placed on an unseeded NGM plate and 5 μL spot of M9 was placed in the direction of the worm’s motion. Individual worms were watched. Measurement of the transition time was started when the worm had reached the M9 droplet edge. The timer was stopped once the full body of the worm was located in the M9 droplet and exhibited C-shaped swimming.

### 2.6. Quantification of DA, 5-HT, and AGE Adducts

Immediately following treatment with La (III), Ce (III), Er (III), or Yb (III), 100,000 L1 worms per treatment condition were pelleted. The supernatant was removed, and the pellet was suspended in 100 μL S Basal and frozen in liquid nitrogen. Pellets were thawed and frozen three times followed by sonication. Samples were centrifuged, and the supernatant was used for quantification of DA or 5-HT using a DA ELISA kit, 5-HT ELISA kit (both from Eagle Biosciences, Amherst, NH, USA) or OxiSelect™ Advanced Glycation End Product (AGE) ELISA kit (Cell Biolabs, San Diego, CA, USA), according to the manufacturer’s instructions. Neurotransmitter and AGE adduct data were normalized to protein content using the BCA assay.

### 2.7. Intracellular Reactive Oxygen Species Determination

Intracellular reactive oxygen species (ROS) were measured using 2,7-dichlorodihydrofluorescein diacetate (DCFD), as previously described [53]. Briefly, 500 L1 worms treated with La (III), Ce (III), Er (III), or Yb (III) were loaded onto a black 96-well plate and treated with 25 µM 2′,7′-dichlorodihydrofluorescein diacetate (DCFDA). Green fluorescence (excitation 490 nm and emission 520 nm) was read immediately and subsequently every 30 min for 6 h.

### 2.8. Quantification of GSH and ATP

*Glutathione Quantification*. Total intracellular glutathione (GSH) levels were measured 24 h post-lanthanide treatment by the 5,5′-dithiobis-2-nitrobenzoic acid–GSH disulfide reductase recycling method, as previously described [54] in whole worm extracts from 30,000 treated worms. ATP production was measured 24 h post-lanthanide treatment using the StayBrite Highly Stable ATP Bioluminescence Assay Kit (BioVision, Milpitas, CA, USA), according to manufacturer’s instructions. ATP and GSH levels were normalized to the protein content of the lysates using the BCA assay.

### 2.9. Oxidative Stress Reporter Assay

Activation of the antioxidant response element was measured using the VP596 strain, which expresses GFP under the control of the antioxidant response element (ARE) promoter for GSH S transferase 4 (*gst-4*) gene. The *gst-4* gene is under control of SKN-1, the worm homolog of nuclear factor (erythroid-derived-2)-like 2 (Nrf2), a transcription factor that responds to environmental insults and binds the ARE to transcribe antioxidant genes. VP596 worms also express RFP under the *dop-3* promoter. L1 VP596 worms were treated with La (III), Ce (III), Er (III), or Yb (III) for 30 min, washed, and transferred to a 96-well plate. The levels of RFP and GFP florescence were measured (RFP: excitation 544 nm and emission 590 nm; GFP: excitation 485 nm and emission 520 nm). Antioxidant response element activity was represented as GFP florescence divided by RFP florescence (normalization to worm number).

### 2.10. Lifespan Analysis

L1 N2 worms were treated with La (III), Ce (III), Er (III), or Yb (III) and aged to L4 stage. Healthy-looking, treated worms (40 per treatment, in duplicate) were then transferred to new OP50-seeded NGM plates. Worms were counted for survival daily and transferred to new plates every 2 days for feeding on fresh OP50. Survival was fitted to a Kaplan–Meier survival curve. Mean lifespan was calculated using Prism 8 software, and log-rank test was performed.

### 2.11. Statistics

Statistical analyses were performed using Prism 8 software (Graphpad, San Diego, CA, USA). Data were tested for normal distribution using the Shapiro–Wilk test, all of which had *p* values indicating normality. Statistical analysis of significance was carried out either by Student’s *t*-test for LD_50_ value comparisons or two-way analysis of variance (ANOVA) for all other data. Log-rank test was used for lifespan analysis. Values of *p* < 0.05 were considered statistically significant.

## 3. Results

### 3.1. Toxicity of Lanthanides to C. elegans

**Figure 1:**
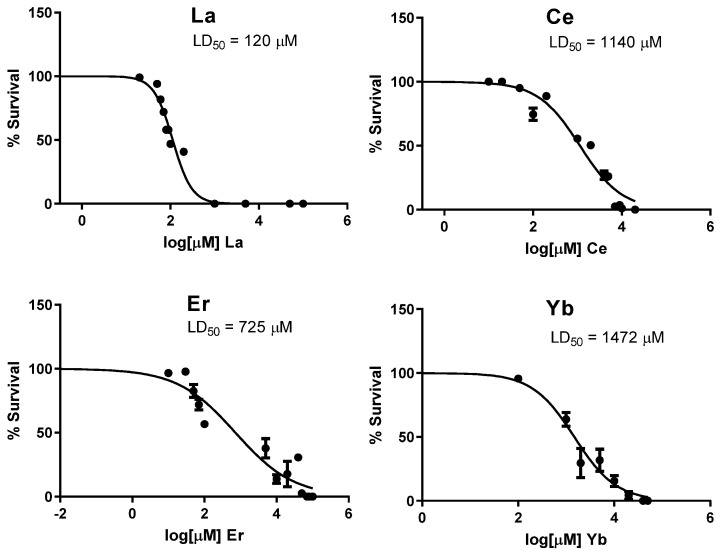
Lanthanide-induced toxicity in *C. elegans*. L1-stage N2 worms were treated with increasing concentrations of (**top-left**) LaCl_3_, (**top-right**) CeCl_2_, (**bottom-left**) ErCl_3_, or (**bottom-right**) YbCl_3_ for 30 min, washed, and transferred to agar plate supplemented with OP50 *Escherichia coli*. Survival was manually counted 48 h post-exposure and fit to a non-linear sigmoidal dose response. LD50 values were calculated and reported. Data points represent the mean + SEM from 5 independent experiments.

While the toxicity of light lanthanides such as lanthanum (La) and cerium (Ce) have been investigated in *C. elegans* before, the toxicity of heavier lanthanides has not been well documented. We selected two heavy lanthanide elements for investigation, erbium (Er) and ytterbium (Yb). Wild-type N2 worms were exposed to increasing concentrations of LaCl_3_, CeCl_2_, ErCl_3_, or YbCl_3_, and dose–response survival curves were generated (Figure 1). The dose required to kill 50% of the population, LD_50_, was calculated from each curve and is presented in Figure 1.

From these data, it is apparent that La (III) was the most toxic to the worms, followed by Er (III), Ce (III), and Yb (III). From the four tested lanthanides, there did not appear to be a trend in LD_50_ values for increasing atomic weight of the lanthanide element. From the dose–response survival curves, we selected doses for further experiments that resulted in under 10% lethality.

### 3.2. Lanthanides Inhibit Dopamine-Dependent Behaviors

**Figure 2:**
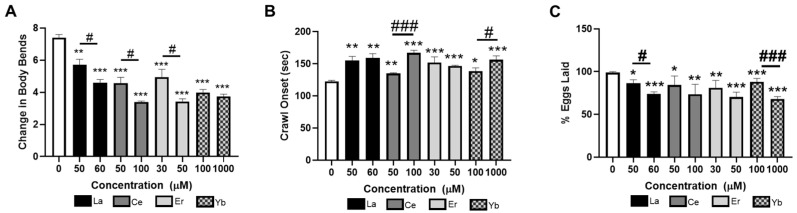
Lanthanides decrease DAergic function in *C. elegans*. L1-stage N2 worms were treated with lanthanides for 30 min, washed, and maintained on NGM agar plates seeded with OP50 *E.* coli. A total of 72 h post-exposure, worms were analyzed for the (**A**) BSR or (**B**) swim-to-crawl transition. (**C**) Alternatively, 30 h after reaching the L4 stage, eggs were quantified on agar or unlaid inside the worm. Data are presented as average (**A**) change in body bends on vs. off *E. coli*, (**B**) time to crawl, or (**C**) % eggs laid ± SEM of five independent experiments. * *p* < 0.05, ** *p* < 0.01, and *** *p* < 0.001 as compared to untreated control. Horizontal bars represent comparisons between concentrations of lanthanide-treated worms. # *p* < 0.05 and ### *p* < 0.001 as compared between lower and higher concentrations of lanthanides.

As exposure to heavy metals, such as MeHg, Mn, and Fe, is associated with damage to the DAergic nervous system in mammals and nematodes, we were interested in whether the selected lanthanides could alter DA-dependent behaviors in *C. elegans*. In *C. elegans*, DA is involved in sensing and altering the rate of locomotion, in response to food and chemicals. The basal slowing response (BSR) measures the change in forward-directed body bends when worms are on or off agar plates containing the *E. coli* food source. Healthy adult N2 worms, with a functioning DAergic system, slow their motion on NGM agar plates spread with *E. coli* as compared to the NGM plates unseeded with bacteria [48]. Worms were treated with La (III), Ce (III), Er (III), or Yb (III) and plated on NGM plates, and BSR was assessed 72 h later upon reaching adulthood. In Figure 2A, all concentrations of La (III), Ce (III), Er (III), and Yb (III) decreased the BSR as compared to the untreated control worms, suggesting damage to the DAergic system. Furthermore, there was significant dose-dependent loss of BSR in response to La (III), Ce (III), and Er (III). To further confirm loss of DAergic function, adult worms were assayed for the swim-to-crawl transition. Worms move in either a sinusoidal crawl or a C-shaped swim. Transitioning between swimming and crawling is controlled by the DAergic nervous system, whereas the transition between crawling and swimming is controlled by the serotonergic system [49]. Seventy-two hours post-lanthanide treatment, worms were placed in a small droplet of M9 on an unseeded NGM plate and the time for the transition between swimming and crawling on the agar was measured. Exposure to all concentrations of lanthanides increased the time it took for the worms to transition between swimming and crawling (Figure 2B), suggesting damage to their DAergic system. Dose-dependent decrements were seen in worms treated with 50 vs. 100 μM Ce (III) or 10 vs. 1000 μM Yb (III). Lastly, worms were assayed for egg laying. The egg-laying behavior is representative of both DA and 5-HT neuronal function. Eggs were quantified from both inside the treated worms and eggs that were laid on the plate, and the percentage of the total eggs that were laid was calculated. Lanthanide treatment significantly reduced the number of eggs laid by the worms (Figure 2C). Dose-dependent effects on egg laying were observed for worms treated with 50 vs. 60 μM La (III) and 100 vs. 1000 μM Yb (III). Taken together, our data suggest that both heavy- and light-molecular-weight lanthanide metals can cause deficits in DAergic function.

### 3.3. Lanthanides Inhibit 5-HT-Dependent Behaviors

**Figure 3:**
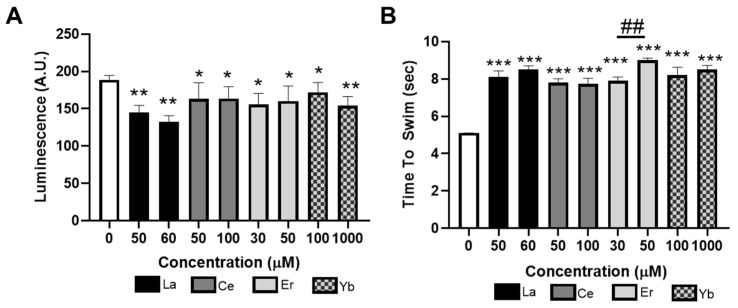
Lanthanides decrease serotonergic function in *C. elegans*. L1-stage (**A**) PE254 or (**B**) N2 worms were treated with lanthanides for 30 min, washed, and maintained on NGM agar plates seeded with OP50 *E. coli*. A total of 72 h post-exposure, worms were analyzed for (**A**) pharynx pump rate or (**B**) crawl-to-swim transition. Data are presented as average (**A**) rate of luminescence for 1 h and (**B**) time to swim ± SEM of five independent experiments. * *p* < 0.05, ** *p* < 0.01, and *** *p* < 0.001 as compared to untreated control. Horizontal bars represent comparisons between concentrations of lanthanide-treated worms. ## *p* < 0.01 as compared between lower and higher concentrations of lanthanides.

As egg laying is controlled by both DA and 5-HT and we found that the lanthanide metals decreased the number of eggs laid by the worms, we were interested in if the lanthanides altered 5-HT-specific behaviors in worms. Two specific behaviors that reflect 5-HT function were analyzed in worms. Pharynx pump rate was assessed in PE254 worms, which express luciferase in their pharyngeal muscles. PE254 worms luminesce as they feed on D-luciferin, and the rate at which the worms luminesce correlates with pharyngeal pumping and 5-HT signaling [52]. L1-stage PE254 worms were treated with La (III), Ce (III), Er (III), or Yb (III) for 30 min and then plated on NGM plates, and the pharyngeal pump rate was assessed 72 h later. Treatment with La (III), Ce (III), Er (III), or Yb (III) significantly reduced the pharyngeal pump rate as compared to untreated worms (Figure 3A). As a second measure of 5-HT-mediated behavior, we measured the transition from crawling to swimming behavior. Seventy-two hours post-lanthanide treatment, the N2 worms were placed on an unseeded NGM plate and 5 μL spot of M9 was placed in the direction of the worms’ motion. The time for transition between crawling and swimming on the agar was measured. As shown in Figure 3B, treatment with lanthanides greatly increases the time it takes for worms to transition from crawling to swimming. Taken together, these data suggest that the serotonergic system is negatively affected by lanthanides in worms.

### 3.4. Lanthanides Decrease Dopamine and Serotonin Contents

**Figure 4:**
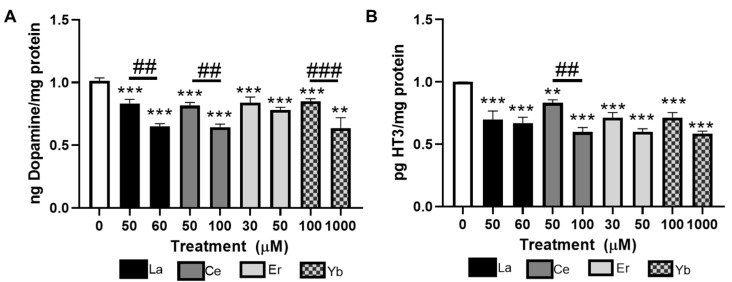
Lanthanides decrease dopamine and serotonin in *C. elegans*. (**A**) DA or (**B**) 5-HT was quantified immediately following exposure to La (III), Ce (III), Er (III), or Yb (III). Data are expressed as means ± SEM of five independent experiments. ** *p* < 0.01, and *** *p* < 0.001 as compared to untreated control. Horizontal bars represent comparisons between concentrations of lanthanide-treated worms. ## *p* < 0.01 and ### *p* < 0.001 as compared between lower and higher concentrations of lanthanides.

As DA- and 5-HT-dependent behaviors in worms were reduced in response to lanthanide treatment, we next quantified the DA and 5-HT contents in extracts of N2 worms treated with lanthanides. Treatment with La (III), Ce (III), or Yb (III) dose-dependently decreased the amount of DA in the worm as compared to the untreated control worms (Figure 4A). Both concentrations of Er (III) also significantly decreased the DA content as compared to the untreated control worms; however, there was not a significant difference between the two doses of Er (III). Treatment with La (III), Ce (III), Er (III), or Yb (III) significantly decreased the 5-HT content in the worms as compared to the untreated control worms (Figure 4B). Of the four lanthanides tested, only Ce had dose-dependent reductions in 5-HT. These data suggest that the decreased DA- and 5-HT-dependent behaviors are reflective of decreased neurotransmitter levels in the worms following exposure.

### 3.5. Lanthanides Induce Oxidative Stress

**Figure 5:**
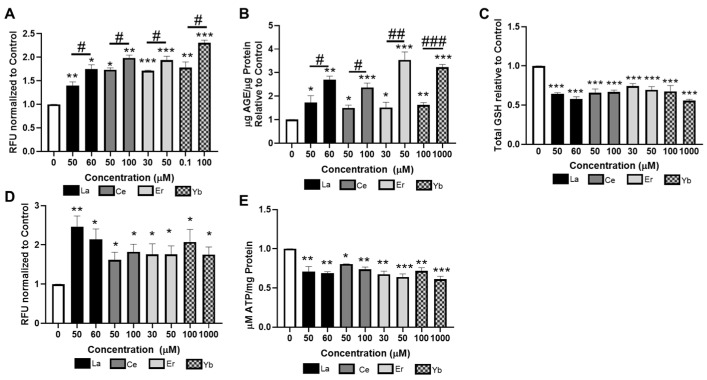
Lanthanides induce oxidative stress in *C. elegans*. Worms were treated for 30 min with La (III), Ce (III), Er (III), or Yb (III). Measures of oxidative stress were either assessed immediately (**A**) or 24 h following exposure (**B**–**E**). (**A**) ROS levels were assessed by DCFDA fluorescence. Data are expressed as mean fluorescence ± SEM for five independent experiments (**B**) AGE protein adducts were measured and normalized to protein content. Data are expressed as means ± SEM from 5 independent experiments. (**C**) Total GSH levels were quantified and normalized to protein content. Data are expressed as mean ± SEM from five independent experiments. (**D**) Quantification of GFP fluorescence of VP596 transgenic worms. Data are expressed as mean fluorescence ± SEM from 5 independent experiments. (**E**) ATP levels were quantified and normalized to protein content. Data are expressed as mean ± SEM from five independent experiments. * *p* < 0.05, ** *p* < 0.01, and *** *p* < 0.001 as compared with untreated control. Horizontal bars represent comparisons between concentrations of lanthanide-treated worms. # *p* < 0.05, ## *p* < 0.01, and ### *p* < 0.001 as compared between lower and higher concentrations of lanthanides.

Oxidative stress is a common consequence of metal exposure. We therefore assessed whether lanthanides could cause oxidative stress in nematodes. Intracellular ROS was measured in worms treated with La (III), Ce (III), Er (III), or Yb (III) by DCFDA staining. Worms treated with lanthanides had significantly higher amounts of ROS generation than the untreated worms (Figure 5A). For all lanthanides tested, there were dose-dependent increases in ROS generation in the treated worms. These data suggest that the treatment of lanthanides induce ROS, which can lead to oxidative stress. ROS can oxidize biomacromolecules, such as DNA, protein, and carbohydrates. Advanced glycation end products (AGEs) are a heterogeneous group of compounds that are formed via the Maillard reaction, which occurs when a reducing sugar reacts in a non-enzymatic way with amino acids in proteins, lipids, or DNA. ROS and metal exposure increase AGE formation, which in turn can drive further ROS generation, creating a vicious cycle [55,56,57]. Lanthanide treatment dose-dependently increased AGE formation in worms as compared to the untreated control worms (Figure 5B). These data suggest that the ROS can cause damage to carbohydrates and proteins in response to lanthanide treatment.

We next investigated antioxidant responses to lanthanide treatments in worms. GSH is the main intracellular thiol that maintains the redox environment of the cell and readily bind to metals via its thiol group. Treatment of *C. elegans* with lanthanides significantly decreased the amount of total GSH inside the worms (Figure 5C). Next, we examined if worms mounted an antioxidant response through SKN-1 (worm homog to Nrf2 (nuclear factor erythroid 2-related factor 2). Nrf2/SKN-1 is a transcription factor that upregulates phase II metabolic and antioxidant genes in response to oxidative stress and xenobiotics. The VP596 strain expresses GFP under the control of the promoter for the SKN-1 target gene *gst-4*. Treatment of La (III), Ce (III), Er (III), or Yb (III) significantly increased GFP fluorescence above the untreated control worms (Figure 5D), indicating that there was increased activation of the SKN-1 response in the worms.

Lastly, we evaluated if the oxidative stress derived from lanthanide exposure could damage the mitochondria. Mitochondrial damage is a common feature in neurotoxicity and has been implicated in neurological conditions, including Parkinson’s disease. As a measure of mitochondrial function, we assessed the ATP levels following lanthanide exposure. Treatment of *C. elegans* with La (III), Ce (III), Er (III), or Yb (III) significantly decreased the ATP content of the worms (Figure 5E). This suggests that there was damage to the mitochondria resulting in less ATP being formed through oxidative phosphorylation.

### 3.6. Lanthanide Treatment Shortens Lifespan

The free-radical theory of aging suggests that the accumulation of oxidative stress is a major determinant of an organism’s lifespan. Both environmental stressors and genetic mutations have shown direct links between oxidative stress and a shorter lifespan [58,59,60]. Worms treated with 60 μM La (III), 100 μM Ce (III), 50 μM Er (III), or 1000 μM Yb (III) for 30 min and 40 worms per treatment were maintained on OP50 *E. coli*-seeded agar plates. The number of living worms was counted daily until all worms died. Kaplan–Meier survival curves were generated, and a log-rank test was performed. Worms treated with lanthanides had significantly reduced lifespans as compared to the untreated worms (Table 1). These data suggest lanthanide-induced oxidative stress has functional consequences in *C. elegans* resulting in a shorter lifespan.

## 4. Discussion

Lanthanide elements are heavy metals that are being incorporated more into consumer goods and are being released increasingly into our environment. Lanthanides released from industrial sources have significantly increased levels of these metals in air and water [6,7,8,9]. It has been estimated that in Europe, the release of lanthanum alone is 50 times higher than the highly regulated toxic metals mercury and cadmium combined [61]. Lanthanides are being found in drinking water, fountain drinks, and other beverages post-waste water treatment [62]. The ability of lanthanides to be neurotoxic has come from reports of lanthanide-containing pharmaceuticals, lanthanum carbonate, and Gd-based contrast agents (GBCAs). Both lanthanum carbonate and GBCAs have been shown to cause encephalopathy, as well as confusion, aphasia, rigidity, and visual disturbances [15,16,17,18,20,21,22]. Likewise, in rodents, La and Ce have been shown to be neurotoxic. La impairs learning, cognition, and memory [23,24,25,26,27]. Similar effects on behavior and memory have been observed in mice exposed to Ce [29,30,31,32]. With the exception of GBCAs, the neurotoxicity of many of the larger-atomic-weight lanthanide series elements are not well characterized.

From rodent, zebrafish, and human cell-line studies, it has become apparent that both DA and 5-HT signaling are impaired by lanthanides. Adult mice exposed to CeCl_3_ for 60 days had decreased neurotransmitters levels, in particular DA [32]. DA and noradrenaline release were shown to be significantly decreased in rat brain synaptosomes exposed to La [33]. Rats exposed in utero to La had significantly decreased levels of 5-HT and acetylcholine, but increased levels of glutamate in the brain [25]. 5-HT levels in the intestine were decreased in zebrafish exposed to cerium oxide nanoparticles, which was due to the ability of cerium oxide nanoparticles to complex with 5-HT [31]. It is unknown if only cerium oxide nanoparticles can form complexes with 5-HT or if other lanthanide oxides or elemental lanthanides can also form these complexes. Negative effects on 5-HT signaling in zebrafish were also observed following La and Praseodymium (Pr) exposures [63]. Complementarily, DAergic, cholinergic, and serotonergic signaling was decreased in larval zebrafish exposed to TbPO_4_ [64]. These fish also had altered swim behaviors including hyperactivity. DA has been shown to bind to several different engineered nanoparticles that contain lanthanides, including Ce, Eu, and Gd [65,66,67,68]. Some of these nanoparticles are being investigated for the detection or imaging of dopamine in vivo. Caution needs to be used as DA can be oxidized by reacting with these nanoparticles and free DA levels can be significantly decreased in aqueous solutions by sequestration of DA-bound nanoparticles [65]. Our data in *C. elegans* also describe how La (III), Ce (III), Er (III), and Yb (III) can cause a reduction in both DA and 5-HT neurotransmitter levels and that functional consequences on behaviors can be observed.

5-HT is an important neurotransmitter in both the central and peripheral nervous systems. In the brain, 5-HT regulates mood. At normal 5-HT levels, people feel focused, emotionally stable, happy, and calm. Additional roles for 5-HT include sleep regulation, healing, and digestion. Serotonergic dysfunction is associated with multiple conditions, including depression, generalized anxiety disorder, and post-traumatic stress disorder (PTSD) [69,70,71,72,73]. In our study, we observed a 20–40% reduction in 5-HT levels in worms treated with La (III), Ce (III), Er (III), or Yb (III). This correlated with a 20–50% reduction in serotonergic-specific behaviors in the worms. 5-HT modulation plays a major role in both the pathophysiology and treatments of anxiety, depression, and PTSD. As lanthanides alter 5-HT signaling, it is possible that people who are exposed to lanthanides are more susceptible to developing PTSD, anxiety disorders, or depression. Interestingly, in rats exposed to perinatal GBCAs, there was increased anxiety-like behaviors and disrupted motor function observed upon adulthood [74]. Selective 5-HT reuptake inhibitors (SSRIs) are a class of pharmaceuticals that inhibit the reuptake of 5-HT from synapses, allowing for prolonged activation of 5-HT receptors by the neurotransmitter. Increasing 5-HT levels in the brain through SSRIs is first-line treatment for depression, anxiety, and PTSD [75,76]. Our data suggest that lanthanide exposure may interfere with the ability of SSRIs to increase 5-HT levels.

Likewise, alterations in DA are the hallmark of Parkinson’s disease (PD). DA regulates mood, learning, memory, and movement. Additional roles for DA include sleep regulation, motivation, and pleasure. PD is a neurodegenerative disorder characterized by the slow progression and irreversible loss of >80% of the nigrostriatal DAergic neurons [77,78,79]. Symptoms of PD involve motor skills and speech impairment. Motor symptoms include muscle rigidity, tremor, slowing of physical movement (bradykinesia), and postural instability. Decreased DAergic stimulation of the motor cortex by the basal ganglia is responsible for these motor effects. Secondary symptoms may include high-level cognitive dysfunction and subtle language problems. Dementia associated with PD has been linked to the accumulation of aggregates termed Lewy bodies [80,81]. Lewy bodies are composed primarily of α-synuclein fibrils, as well as other proteins, lipids, and membranous organelles. It is estimated that around 10% of PD cases are genetic in origin, suggesting a large role for environmental agents in the disease’s etiology. Exposure to metals such as mercury, manganese, iron, and copper has been associated with the development of PD. These metals have been shown to decrease DAergic signaling as well as accumulate in regions of Lewy bodies in human, rodent, and worm models, as well as in vitro in cell cultures [82,83,84,85]. In our study, we observed a 20–40% reduction in DA levels in worms treated with La (III), Ce (III), Er (III), or Yb (III). This correlated with a 20–50% reduction in DAergic-specific behaviors in the worms. Currently, it is unknown whether exposure to lanthanides is a risk factor for developing PD. However, it has been observed that rates of overlapping neuropathologies, such as PD and Alzheimer’s disease, are increasing in urban areas where there is exposure to mixed-metal-containing nanoparticulates during the first two decades of life [86]. Some of the metals implicated were the lanthanides La, Ce, and Pr [86]. Alternatively, CeO_3_ nanoparticles are being investigated as a PD therapeutic. CeO_3_ nanoparticles have been shown to improve locomotor activity in a haloperidol-induced PD rat model and prevent α-synuclein fibrilization in vitro [87,88,89]. These discrepancies suggest that there might be differential effects of lanthanides on the DAergic system that are species- or developmental stage-dependent. Further investigation is needed to elucidate the role of lanthanides in the development of PD.

A common feature to neurological conditions involving DA dysregulation is the presence of oxidative stress. DA is a reactive molecule that can be oxidized to ROS-generating DA quinone and semiquinone radicals [90,91]. Likewise, oxidative stress is a proposed mechanism for the observed neurotoxicity of lanthanide metals. Residents who live near lanthanide-contaminated sites have elevated levels of serum lanthanides and oxidative stress biomarkers, such as malondialdehyde and 8-isoprstane [92]. Children exposed in utero to mixtures of REEs, including lanthanides, had damaged mitochondria [93]. Additionally, exposure to REEs during pregnancy has been associated with premature rupturing of membranes [94], which arise from oxidative damage to the proteins and lipids that comprise the birth membranes. Mice exposed to LaCl_3_ showed increased microglia activation and increased inflammatory and oxidative stress markers [23].

Previous studies have explored oxidative stress mediated by La and Ce in *C. elegans* using a diverse array of experimental conditions. Lan et al. exposed *C. elegans* to doses of La, Ce, or Ce + La in the agar from early adulthood and subsequently for 10 days [95]. This models adult chronic exposure as the worms were treated for nearly half of their typical lifespan. La, Ce, or the La + Ce mixture induced oxidative stress and reduced the worm’s lifespan [95]. In our study, we treated the worms with an acute dose of lanthanide early in the L1 life stage and assessed both behavioral and oxidative stress markers later in life. We report that the exposure of *C. elegans* to LaCl_3_, CeCl_3_, ErCl_3,_ or YbCl_3_ increased ROS production and the generation of AGE adducts on proteins. Interestingly, Han et al. compared ROS generation in L1 and L4 worms exposed to La(NO_3_)_3_ and found significant ROS production in the exposed L4s and not in the L1s, even though the L1s were more sensitive to La(NO_3_)_3_ [36]. Differences in the L1 response to La between this study and ours may due to the salt of La being different, LaCl_3_ vs. La(NO_3_)_3_. This suggests that the anion in the salt may have effects on ROS production following La exposure. We also found that treatment with lanthanide metals decreased GSH content and caused an activation of the ARE. These data are in agreement with mouse studies where La exposure in mice has been shown to activate the antioxidant transcription factor Nrf2 in the choroid plexus [96], the area that is implicated in lanthanides entering the brain [19]. As REEs have been shown to damage mitochondria [93] and mitochondrial damage is implicated in many neurological disorders, including Parkinson’s disease, it is imperative to further investigate whether mitochondrial damage induced by lanthanides contributes to lanthanide-induced neurological dysfunction.

While there have been epidemiologic data in human populations and experimental data from rodents and cell lines for low-atomic-mass lanthanides, such as La and Ce, there has not been extensive study of larger lanthanides. We report that Er and Yb elicited equal amounts or higher of ROS and oxidative stress markers in *C. elegans* as La (III) and Ce (III). Inhalation studies of lanthanides containing nanoparticles have reported increased levels of lipid peroxidation end product malondialdehyde as well as significantly decreased levels of glutathione (GSH) in rodents exposed to YbO_3_ nanoparticles [97]. Similarly, exposure of Hep-G2 liver cells to ErO_3_ nanoparticles increased intracellular reactive oxygen species (ROS) and DNA damage and induced apoptosis [98,99]. From these previous studies, it was not apparent whether the oxidative stress arose from the lanthanide element, exposure to nanoparticulate matter, or both. Arnold et al. have described how CeO_2_ nanoparticles are more toxic to *C. elegans* than dissolved non-nanoparticulate CeO_2_ powder [100]. Furthermore, it has also been reported that coatings of CeO_2_ nanoparticles influence toxicity in *C. elegans* [101]. These data suggest that lanthanides that exist as nanoparticulates can behave differently than non-nanoparticulate forms of lanthanides. Our data using Er and Yb (III) chloride salts support that both Er (III) and Yb (III) can cause oxidative stress and toxicities both as a salt and as a nanoparticle.

## 5. Conclusions

Taken together, our data suggest that early-life exposure to either the low-atomic-number (La (III) and Ce (III)) and high-atomic-number (Er (III) and Yb (III)) lanthanides can cause DAergic and serotonergic dysfunctions in *C. elegans*. These neurological deficits were found to comprise both reduced levels of neurotransmitters and altered behavioral functions. Concurrent with the neurological dysfunction, there were increased levels of ROS, oxidative stress, and reduced lifespan following exposure to the four lanthanides investigated. These data suggest that lanthanide elements may be contributors of diseases of DA and 5-HT dysfunction, such as PD, anxiety, depression, and PTSD.

## Figures and Tables

**Table 1:** Mean lifespan following lanthanide treatment.

Metal	Mean Lifespan (Days)	*p* Value
Untreated	21	-
60 mM La (III)	15.5	<0.0001
100 mM Ce (III)	14	<0.0001
50 mM Er (III)	13	<0.0001
1000 mM Yb (III)	14	<0.0001

## Data Availability

The data that support the findings of this study are available from the corresponding author upon reasonable request.

## Abbreviations

5-HT, serotonin; ANOVA, analysis of variance; AGE, advanced glycation end products; ARE, antioxidant response element; BBB, blood–brain barrier; BSR, basal slowing response; CSF, cerebrospinal fluid; DA, dopamine; DAergic, dopaminergic; DCFDA, 2′,7′-Dichlorodihydrofluorescein diacetate; e-cigarettes, electronic cigarettes; ELISA, enzyme-linked immunosorbent assay; GBCA, gadolinium-based contrast agent; GFP, green fluorescent protein; GSH, glutathione; Nrf2, nuclear factor (erythroid-derived-2)-like 2; RRE, rare earth element; ROS, reactive oxygen species; SEM, standard error of the mean.
